# The developing story of Sprouty and cancer

**DOI:** 10.1007/s10555-014-9497-1

**Published:** 2014-04-18

**Authors:** Samar Masoumi-Moghaddam, Afshin Amini, David Lawson Morris

**Affiliations:** UNSW Department of Surgery, University of New South Wales, St George Hospital, Kogarah, Sydney, NSW 2217 Australia

**Keywords:** Sprouty, Cancer, MAPK/ERK, RTK, Growth factors

## Abstract

Sprouty proteins are evolutionarily conserved modulators of MAPK/ERK pathway. Through interacting with an increasing number of effectors, mediators, and regulators with ultimate influence on multiple targets within or beyond ERK, Sprouty orchestrates a complex, multilayered regulatory system and mediates a crosstalk among different signaling pathways for a coordinated cellular response. As such, Sprouty has been implicated in various developmental and physiological processes. Evidence shows that ERK is aberrantly activated in malignant conditions. Accordingly, Sprouty deregulation has been reported in different cancer types and shown to impact cancer development, progression, and metastasis. In this article, we have tried to provide an overview of the current knowledge about the Sprouty physiology and its regulatory functions in health, as well as an updated review of the Sprouty status in cancer. Putative implications of Sprouty in cancer biology, their clinical relevance, and their proposed applications are also revisited. As a developing story, however, role of Sprouty in cancer remains to be further elucidated.

## Introduction

Mitogen-activated protein kinase (MAPK) signaling pathways are among the most widespread regulatory mechanisms of the eukaryotic cell biology. The first mammalian MAPK pathway to be identified and entirely mapped is extracellular signal-regulated kinase or MAPK/ERK (hereafter ERK). ERK orchestrates a signal transduction from cell membrane molecules to the transcriptional machinery to promote cell growth, differentiation, and survival. As with other MAPKs, ERK represents a three-tiered kinase cascade composed of the sequentially-acting kinases. ERK is activated by a wide range of extracellular signals including growth factors, cytokines, hormones, and neurotransmitters. Signal transduction is initiated when a ligand binds its transmembrane receptor tyrosine kinase (RTK) and thereby activates Ras, a small G protein anchored to the plasma membrane. Ras subsequently recruits from the cytosol to the cell membrane and activates Raf serine/threonine-specific kinases of MAPK-kinase kinase (MAP3K) family. Through serine/threonine phosphorylation, Raf activates a family of dual specificity kinases known as MAPK kinases (MAP2K) or MAPK/ERK kinases (MEKs). By concomitant tyrosine and threonine phosphorylation, MEKs activate MAPK (Erk). Phosphorylated Erk eventually induces gene expression by direct and indirect targeting of transcription factors. To setup a biologically coordinated infrastructure for physiologically appropriate outcomes, ERK and its core modules are under tight, multilayered control of positive and negative regulators, including the Sprouty protein family.

Sprouty was discovered by Hacohen et al. who initially described it as a common antagonist of fibroblast growth factor (FGF) and epidermal growth factor (EGF) signaling pathways in Drosophila [1, 2]. In a search of the Expressed Sequence Tag (EST) database, they identified three human homologs of the fly gene designated *hSpry1-3* [1]. The fourth mammalian homolog, *hSpry4*, was later discovered in mice [3] and humans [4]. Emerging evidence later showed that Sprouty specifically inhibits activation of ERK in response to a wide range of trophic factors, including FGF [5, 6], platelet-derived growth factor (PDGF) [5], vascular endothelial growth factor (VEGF) [6], nerve growth factor (NGF) [7], brain-derived neurotrophic factor (BDNF) [8], and glial cell line-derived neurotrophic factor (GDNF) [9]. The biological functions of the Sprouty proteins have been attributed to its conserved motifs. These mainly include the N-terminal canonical Casitas B-lineage lymphoma (c-Cbl) binding domain (CBD) containing a key tyrosine residue; the serine-rich motif (SRM); and the C-terminal cysteine-rich domain (CRD) also known as the Sprouty (or translocation) domain. Among the Sprouty isoforms, Spry2 exhibits the highest evolutionary conservation, with the human Spry2 showing 97, 85, and 51 % sequence homology, in the CRD domain, to the mouse, chick, and Drosophila protein, respectively [10]. Although Spry2 appears to be ubiquitously expressed in embryonic and adult tissues, the expression of other isoforms shows organ/tissue specificity [4, 10, 11].

The Sprouty proteins are currently recognized as key regulators of ERK signaling that act on different levels of the pathway. Furthermore, they are part of a tightly-orchestrated regulatory mechanism where interactions with a variety of players lay the basis for a crosstalk between ERK and partner cascades. Nevertheless, aberrant activation of ERK and deregulation of Sprouty occurs in a variety of pathological conditions, including malignant transformation. In this article, complex functions of the Sprouty family under physiological conditions are revisited, altered expression of Sprouty in different cancer types and its impact on cancer development, progression, and metastasis studied by different investigators are reviewed, and clinical application of the deregulated Sprouty as a biological marker and/or a focus of targeted strategies is discussed.

## Sprouty: a versatile modulator with complex functionality

Since discovery of Sprouty in 1998 [1], an expanding body of evidence has continued to support its crucial role in regulation of various physiological processes. Initial studies by Minowada et al. [10] and Tefft et al. [12] revealed that this protein family and its regulatory relationship with FGF-induced signaling are evolutionarily conserved. Through comparative genomic analysis, the linkage between the human Sprouty and FGF genes was later reported [13]. Sprouty regulates tubular morphogenesis as a fundamental process in organogenesis and angiogenesis where FGF signaling is particularly involved [14–16]. Apart from its crucial role in embryogenesis, Sprouty has been implicated in regulation of physiological events in adult organs. Table 1 summarizes a number of studies in which implication of Sprouty in developmental and physiological events has been documented.

**Table 1:** Sprouty implication in developmental and physiological processes reported by some investigators

Investigators	Sprouty isoform	Developmental/Adult physiological event
Hacohen et al. [1]	dSpry	Tracheal development
Kramer et al. [2]	dSpry	Eye development
Minowada et al. [10]	mSpry2 and 4	Limb development
Tefft et al. [12]	mSpry2	Lung development
Furthauer et al. [17]	zSpry4	Midbrain development
Zhang et al. [18]	mSpry1, 2 and 4	Craniofacial and trunk development
Gross et al. [19]	mSpry1	Kidney development
Chi et al. [20]	hSpry2	Ureteric branching
Lo et al. [21]	mSpry1 and 2	Breast development in puberty and pregnancy
Anteby et al. [22]	hSpry1, 2, and 3	Placental villi sprouting
Haimov-Kochman et al. [23]	hSpry2	Follicle maturation and corpus luteum formation
Lin et al. [24]	mSpry2	Patterning of midbrain and anterior hindbrain
Shim et al. [25]	mSpry2	Inner ear development
Basson et al. [26]	mSpry1	Ureteric branching
Boros et al. [27]	mSpry1 and 2	Ocular lens development
Chi et al. [28]	mSpry2	Male sex organogenesis
Natanson-Yaron et al. [29]	hSpry2	Placental villi sprouting
Price et al. [30]	hSpry4	Kidney development
Gross et al. [8]	mSpry2	Neuronal differentiation
Shaw et al. [31]	mSpry2	Lung development
Laziz et al. [32]	hSpry1, 2 and 4	Muscle regeneration
Hamel et al. [33]	hSpry2	Oocyte developmental competence
Klein et al. [34]	mSpry4 (**+** mSpry1 or 2)	Growth and development of rodent incisors
Jaggi et al. [35]	mSpry4	Pancreas development
Wang et al. [36]	xSpry1	Gastrulation
Pan et al. [37]	mSpry2	Lens and lacrimal gland development
Purcell et al. [38]	mSpry1 and 2	Temporomandibular Joint development
Sieglitz et al. [39]	dSpry	Neuronal and glial differentiation
Kuracha et al. [40]	mSpry1 and 2	Eyelid closure
Velasco et al. [41]	hSpry2	Endometrial gland developing and branching
Sigurdsson et al. [42]	hSpry2	Breast morphogenesis
Ching et al. [43]	mSpry1 and 2	External genitalia development

*dSpry* Drosophila Sprouty; *hSpry* human Sprouty; *mSpry* mouse Sprouty; *xSpry* Xenopus Sprouty; *zSpry* zebra fish Sprouty

At cellular level, Sprouty modulates key processes including proliferation, differentiation, motility, and survival through regulation of ERK and parallel pathways, as well as interaction with a number of effectors and regulators. As listed in Table 2, various regulatory effects of the Sprouty proteins in normal and neoplastic cells have been documented in literature. The Sprouty-mediated modulation, however, occurs in a cell- and context-dependent manner where a number of facts and factors, as described below, are involved in the determination of the eventual response.

**Table 2:** Responses of different cell types to the Sprouty-induced regulation reported by different investigators

Investigators	Spry	Stimulator	Cell	Response (×, inhibited; ✓,enhanced; U unaffected)								Pathway/Molecule
				P	M	I	D	A	Ap	T	C	
Normal cells												
Impagnatiello et al. [6]	1, 2	FGF, VEGF	HUVEC	**×**			**×**					ERK
		EGF		**×**								Not ERK
Gross et al. [5]	1, 2	FGF, NGF, PDGF	NIH3T3	**×**					U			ERK
Lee et al. [44]	4	FGF, VEGF	HUVEC	**×**	**×**							ERK
Huebert et al. [45]	1	VEGF	CPAE	**×**								ERK
Poppleton et al. [46]	2	Serum	IEC-6		**×**							Rac1 GTPase
				**×**								ERK
Zhang et al. [47]	2	FGF, EGF, PDGF, serum	VSMC	**×**	**×**							N/A
de Alvaro et al. [48]	2	FGF	C2C12	**×**								ERK
							✓					AKT
Tsumura et al. [49]	4		C2C12		**×**							Cofilin
Fong et al. [50]	2 KD	FGF	NIH3T3							✓	✓	ERK
Wang et al. [51]	4	-	HUVEC	**×**								N/A
Sutterlüty et al. [52]	2	Serum	WI38	**×**	**×**							ERK
Ding et al. [53]	2 DR	TGFβ1	NIH3T3	✓								TGFβ1/Smad
Ding et al. [54]	2 DR	TNF-α	NIH3T3 MLE15						**×**			TNF-α/P38 MAPK
Lito et al. [55]	2	–	MSU1.1						**×**			AKT, HDM2, p53
Tennis et al. [56]	4	–	B2B	**×**	**×**	**×**				✓^ME^		Wnt7A/Fzd9/PPARγ
Jung et al. [57]	1 KD	FGF4	J1-mESCs				✓					ERK
Sigurdsson et al. [42]	2 KD	–	D492	U	✓					✓^EM^	✓	EGFR
Felfly et al. [58]	2 KD	–	hESC	**×**			U		✓			N/A
	4 KD			U					**×**			
	2 KD	FGF, EGF		✓								
	4 KD			U								
Mekkawy et al. [59]	1	uPA, EGF	HEK293		**×**							N/A
Neoplastic cells												
Gross et al. [5]	1, 2	NGF, FGF	PC12				**×**					N/A
Sasaki et al. [60]	4	NGF, FGF	PC12				**×**					ERK
Yigzaw et al. [61, 62]	2	FGF, EGF, PDGF, serum	HeLa	**×**								RTK
					**×**							PTP1B/p130Cas
Wong et al. [7]	2	EGF	PC12				✓					RTK
		NGF, FGF					**×**					
Lee et al. [63]	2	HGF/SF	SK-LMS1	**×**	**×**	**×**		✓			**×**	c-Met, ERK, AKT
Lo et al. [21]	2 DN	–	MCF-7	✓							✓	N/A
Fong et al. [50]	2	HGF	SNU449	**×**								c-Met, ERK
Wang et al. [51]	4	–	LNCaP, PC3	U								N/A
			DU145		**×**							
Edwin et al. [64]	2	Serum	HeLa	**×**	**×**							AKT, PTEN, Rac1
Ishida et al. [9]	2	GDNF	TGW	**×**			**×**					Ret
Sutterlüty et al. [52]	2	Serum	VL-8	**×**	**×**							ERK
			A-549 ^V/V^ VL-4, VL-2	**×** ^V/V^	**×**							
Edwin and Patel [65]	2 KD	Serum	SW13						✓			AKT, ERK, Cbl
Lee et al. [66]	2	–	Huh7, SNU449	**×**								ERK
Jaggie et al. [35]	4	FGF, Serum	PANC-1		**×**			**×**				PTP1B/p130Cas
Frank et al. [67]	2	–	Nalm-6	**×**					✓			ERK
Lito et al. [55]	2	EGF	PH3MT						**×**			Rac1 GTPase
Tennis et al. [56]	4	–	H157, H2122	**×**	**×**	**×**					**×**	Wnt7A/Fzd9/PPARγ
Holgren et al. [68]	2	HGF, Serum	HCT-116	✓^V/V^	✓	✓			**×** ^V^			c-Met
Barbachano et al. [69]	2	–	SW480-ADH					**×**		**×** ^ME^		E-cadherin
Schaaf et al. [70]	1 KD	–	RD, TE381T	**×**					✓			Ras/ERK
Wang et al. [71]	2 DN	–	HLE	✓ ^V^					U			AKT, ERK, PKM2
Alsina et al. [72]	4	NGF	PC12				**×**					TrkA/ERK, Rac1GTPase
Mekkawy et al. [59]	1	uPA, EGF	Saos-2, MDA-MB-231, HCT116		**×**	**×**						N/A
Vanas et al.	2, 4	Serum	MCF-7, MDA-MB231	**×**							**×**	ERK
					**×**							N/A

*Spry* Sprouty; *P* proliferation; *M* migration; *I* invasion; *D* differentiation; *A* adhesion; *Ap* apoptosis; *T* transition/transformation; *C* colony/foci formation; *N/A* not available; *FGF* fibroblast growth factor; *VEGF* vascular endothelial growth factor; *NGF* nerve growth factor; *EGF* epidermal growth factor; *PDGF* platelet-derived growth factor; *TNF-α* tumor necrosis factor alpha; *HGF/SF* hepatocyte growth factor/scatter factor; *GDNF* glial cell line-derived neurotrophic factor; *DR* downregulated Sprouty; *KD* knocked down Sprouty; *DN* dominant-negative mutant of Sprouty; *HUVEC* human umbilical vein endothelial cells; *NIH3T3* mouse embryonic fibroblast cell line; *CPAE* calf pulmonary artery endothelial cell line; *IEC-6* normal rat small intestine epithelial cell line; *VSMC* vascular smooth muscle cells; *C2C12* mouse myoblast cell line; *WI38* normal human embryonic lung fibroblast cell line; *MSU1.1* human fibroblast cell line; *B2B* human nontransformed lung epithelial cell line; *J1 mESCs* mouse embryonic stem cells; *D492* breast epithelial stem cell line; *hESC* human embryonic stem cells; *HEK293* human embryonic kidney cells; *PC12* a cell line derived from pheochromocytoma of the rat adrenal medulla; *HeLa* human cervical cancer cell line; *SK-LMS-1* human leiomyosarcoma cell line; *MCF-7* breast cance cell line; *LNCaP* human prostate adenocarcinoma cell line; *PC3* human prostate adenocarcinoma cell line; *DU145* human prostate carcinoma cell line; *TGW* human neuroblastoma cell line; *VL-8*, *A-549*, *VL-4*, and *VL-2* non–small cell lung cancer (NSCLC) cell lines; *SW13* adrenal cortex adenocarcinoma cell line; *Huh7* and *SNU449* human hepatocellular carcinoma cell lines; *PANC-1* human pancreatic epithelioid carcinoma cell line; *Nalm-6* pre–B-cell tumor cell line; *PH3MT HRas*-expressing derivative of MSU1.1 human fibroblast cell line; *H157* and *H2122* non–small cell lung cancer (NSCLC) cell lines; *HCT-116* human colon cancer cell line; *SW480-ADH* human colon cancer cell line; *RD* and *TE381T* embryonic rhabdomyosarcoma cell lines; *HLE* human hepatocellular carcinoma cell line

*ME* mesenchymal-epithelial transition; *EM* epithelial-mesenchymal transition; *V/V* both *in vitro* and *in vivo*; *V in vivo*; not otherwise marked *in vitro*

### Cell and context dependency

It is evident that cellular behavior in response to the growth factor stimulation and Sprouty-mediated regulation varies from cell to cell. For example, while Spry2 inhibits the differentiation of PC12 pheochromocytoma cells in response to FGF [5, 7], it promotes FGF-induced differentiation of C2C12 myoblasts [48]. Depending on the cellular context and innate physiological characteristics, different components of the RTK signalosome are activated by different ligands [73]. Moreover, strength and duration of the signal transduction are among critical determinants of cell fate in response to activation and regulation of the RTK signaling [74]. In NIH3T3 fibroblasts and PC12 cells, for example, whereas transient activation of ERK during mid-G1 phase leads to cell cycle progression and hence proliferation in the former, sustained ERK activity induces cell-cycle withdrawal and neuronal differentiation in the latter [75]. Accordingly, Sprouty was shown to inhibit proliferation of NIH3T3 cells and differentiation of PC12 cells in response to growth factor stimulation [5]. It will be discussed in the following sections that how Sprouty modulates the RTK signaling depending on the intersection point where it interferes as well as on its interplay with other interacting molecules.

### Growth factor dependency and pathway sensitivity

Depending on the RTK activated and the downstream pathway(s) affected, Sprouty differentially modulates growth factor actions and thereby elicits divergent responses. Hence, Sprouty isoforms are able to selectively uncouple growth factor-induced signal transductions. In a study by Impagnatiello et al. [6] indicating an anti-proliferative effects of the overexpressed Spry1 and Spry2 on endothelial cells in the presence of FGF, VEGF, and EGF, while FGF- and VEGF-induced activation of ERK were repressed by Sprouty, EGF-activated ERK was left unaffected. It was revealed in a study by Sasaki et al. [60] that the expression of Spry2 and Spry4 in HEK293 cells inhibited FGF-induced ERK signaling but did not affect EGF or PDBu activation of ERK. Conversely, the expression of dominant negative mutants of Spry2 and Spry4 enhanced and prolonged FGF, but not EGF, activation of ERK. They later found that Spry4 suppresses VEGF-induced, Ras-independent activation of Raf1 but does not affect the Ras-dependent cascade induced by EGF [76]. Furthermore, evidence supports a positive feedback loop whereby EGF stimulation of ERK signaling is potentiated and sustained by Sprouty. This paradoxical effect was initially investigated by Wong et al. [7] in PC12 cells employed as a proliferation/differentiation responsive model. It is known that FGF and NGF induce differentiation of PC12 cells into a neuron-like phenotype via sustained activation of ERK, whereas EGF stimulation transiently activates ERK and thus promotes cell proliferation [74, 77]. Wong et al. reported that Spry1 and Spry2 inhibited differentiation of PC12 cells induced by FGF-activated ERK, yet they augmented ERK activity in response to EGF and hence promoted differentiation of PC12 cells [7]. This effect represents an intriguing role of Sprouty in protecting EGF receptor (EGFR), which will be discussed later. Using NIH3T3 and PC12 cells transfected with Spry1 or Spry2, Gross et al. [5] observed that Sprouty restricted cell proliferation and growth factor-induced differentiation, but did not promote apoptosis. The investigators found that Spry1 and Spry2 impeded FGF or PDGF stimulation of ERK, but did not affect phosphatidylinositol 3-kinase (PI3K)/AKT pathway through which many RTK-mediated survival signals are relayed. In another study, De Alvaro et al. indicated that FGF stimulation of C2C12 myoblasts induces proliferation and a differentiation-defective phenotype associated with sustained activation of ERK and lack of activation of AKT. Overexpressed Spry2, however, conferred myogenic differentiation properties that were accompanied by repression of ERK and activation of AKT [48].

### Transcriptional regulation of the Sprouty expression

ERK pathway is known to generally upregulate Sprouty [78]. However, inducibility of the Sprouty isoforms in response to the growth factor stimulation may vary in a cell type- and context-dependent manner. In an initial study by Ozaki et al., the expression of the Sprouty genes, including *Spry1*, was shown to be positively regulated by ERK [78]. Parallel studies consistently reported that the expression of *Spry2* and *Spry4* was rapidly induced by growth factors in fibroblasts [5], endothelial cells [6], and HEK293 cells [60], yet concomitant downregulation of *Spry1* was observed [5, 6]. Moreover, Kral et al. [79] demonstrated that neither growth factor stimulation nor Ras activation increased the Spry1 protein levels in WI38 normal human lung fibroblasts. Since Spry1 in their cell cycle analysis with WI38 cells was constantly expressed, they concluded that mitogenic signaling is not sufficient to modulate the Spry1 expression, and that Spry1, as appeared in earlier studies [19, 80, 81], is more likely modulated by differentiation processes. In agreement, partner pathways and mechanisms, too, have been shown to play a role in modulating transcriptional expression of the Sprouty proteins. Choi et al. [82] indicated that Spry1 is the only Sprouty isoform induced by T cell receptor (TCR) stimulation in murine CD4^+^ T cells and that ensuing expression of Spry1 with dual output impacts TCR signaling depending on their differentiation state. In contrast, Frank et al. observed in mouse splenic B cells that combined activation of CD40 and B cell receptor (BCR), known as a stimulator of B-cell proliferation and survival, induces Spry2, but not Spry1, through an ERK-dependent negative feedback loop which attenuates activation of ERK, thereby implicating Spry2 in regulating antigen-induced expansion of mature B cells [67]. Ding et al. reported that transforming growth factor-beta1 (TGFβ1) signaling downregulates Spry2 protein in Swiss 3 T3 cells in a MAPK-independent manner with possible involvement of Smad pathway [53]. Later, they also implicated tumor necrosis factor-alpha (TNF-α) signaling in the Spry2 downregulation via p38 MAPK led to apoptosis of Swiss 3 T3 fibroblasts and MLE15 lung epithelial cells [54]. Different growth factor isoforms may also variably stimulate the RTK induction of the Sprouty expression. Jiang et al. observed in granulosa cells that while FGF1, FGF4, and FGF8 enhanced the expression of *Spry2* and *Spry4*, and FGF8 additionally increased the abundance of *Spry1* messenger RNA (mRNA), FGF10 and FGF18 failed to induce the Sprouty expression [83, 84]. Moreover, the presence of GC-rich regions in *Spry1* [19], *Spry2* [85], and *Spry4* [11] promoters suggests spatiotemporal regulation of the Sprouty expression by tissue-specific transcription factors. Accordingly, the transcription factors Wilms tumor suppressor 1 (WT1) [19], cAMP response element-binding protein (CREB) and specificity protein 1 (SP1) [8], and peroxisome proliferator-activated receptor gamma (PPARγ) [56] have been implicated in normal development of kidney and central nervous system, as well as in the inhibition of lung tumorigenesis via activating *Spry1*, *Spry2*, and *Spry4* promoters, respectively. Also, Sabatel et al. identified *Spry1* as a target of the angiostatic agent 16K prolactin which was shown to induce NF-kappaB-dependent upregulation of *Spry1* in primary and human endothelial cells. They showed that Spry1 silencing protects endothelial cells from apoptosis and induces endothelial cell adhesion, migration, and tube formation and argued that Spry1 acts as an endogenous inhibitor of angiogenesis [86].

### Modulation of the Sprouty stability by post-translational mechanisms

Apart from transcriptional regulation of the protein expression, intracellular level of Sprouty is post-translationally controlled, as well. Polyubiquitylation and proteasomal degradation of active Sprouty mediated by the E3 ubiquitin ligase c-Cbl is a tyrosine phosphorylation-dependent process that temporally limits the Sprouty intervention [87, 88]. Mason et al. showed that although Spry2/c-Cbl complex formation is dispensable for the inhibitory effect of Spry2 on the FGF-activated ERK, it mediates degradation of Spry2 in response to FGF. Thus, Spry2 accumulates to higher levels and inhibits FGF-induced signaling more efficiently in c-Cbl-null mouse embryonic fibroblasts (MEFs) than in control MEFs [89, 90]. Reporting bimodal expression of Spry2 along with sustained elevation of Spry4 during cell cycle progression, Mayer et al. [91] indicated that second phase in the expression profile of Spry2 as transient attenuation of the protein expression during late G1 is solely dependent on cell cycle-specific ubiquitination by c-Cbl. DaSilva et al. [92] showed that serine phosphorylation on Ser112 and Ser121 by MAPK-interacting kinase 1 (Mnk1) provides Spry2 with balanced phosphorylation of Tyr55 that leads to the protein stabilization. As such, mutation of theses serine residues or inhibition of Mnk1 augmented RTK-mediated phosphorylation of Tyr55, thereby enhancing c-Cbl-mediated degradation of the protein. Edwin et al. [93] reported that the HECT domain-containing E3 ubiquitin ligase Nedd4 binds and polyubiquitinates Spry2 to regulates its cellular content along with its ability to modulate RTK signaling. They found that Nedd4 requires Mnk2-dependent phosphorylation of Ser112/Ser121 for its interaction with Spry2. Another E3 ubiquitin ligase, Seven in Absentia homolog 2 (Siah2), has also been implicated in post-translational regulation of the Sprouty content. Nadeau et al. [94] indicated that coexpression of Siah2 resulted in proteasomal degradation of Spry1, Spry2, and, to a lesser extent, Spry4 in a tyrosine phosphorylation-independent manner. As with c-Cbl, it was shown that RING finger domain of Siah2 binds the N-terminal domain of Spry2 to mediate their interaction. Consistently, it was later reported that a dominant-negative Siah2 RING mutant primarily increased the Sprouty content and activity [95]. Furthermore, it was demonstrated in a study by Ding et al. on Swiss 3 T3 cells that TGFβ1 not only downregulates the expression of Spry2, but also induces the protein degradation via a lysosome-dependent pathway [53]. They concluded that downregulation of Spry2 by TGFβ1 at transcriptional and post-translational levels lays a basis for crosstalk between TGFβ1 signaling and EGF, as well as FGF-induced ERK in mesenchymal cells. Haigal et al. showed that hypoxia increased the Spry4 expression in several cell types through HIF-dependent transcription as well as increased mRNA stability [96]. Furthermore, Anderson et al. [97] demonstrated that prolyl hydroxylation of Spry2 by prolyl hydroxylase domain proteins (PHDs) during normoxia targets it for recognition and ubiquitination by von Hippel-Lindau (pVHL)-associated E3 ubiquitin ligase.

**Fig. 1:**
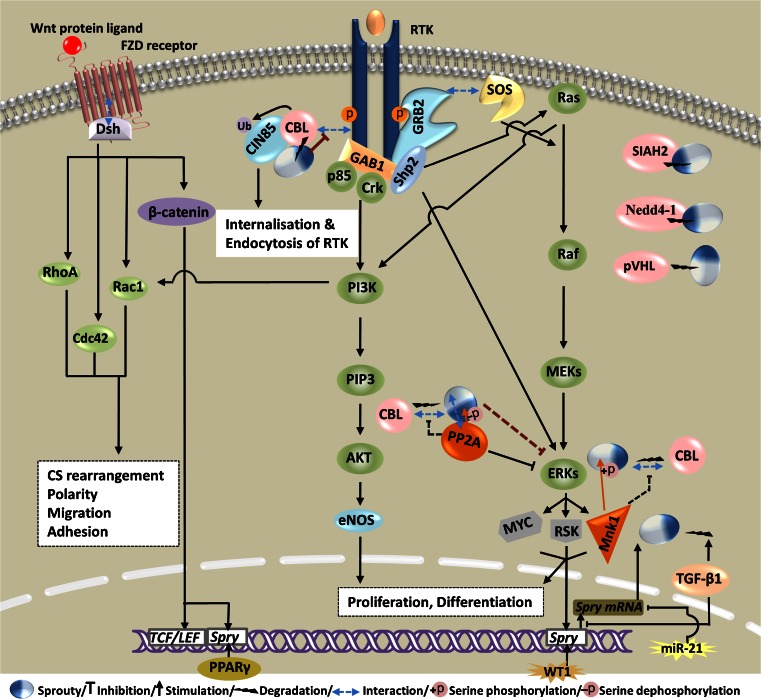
Representative regulators of the Sprouty cellular content at transcriptional and post-translational level irrespective of the Sprouty isoform and cell type. *MAPK/ERK* is the main pathway to upregulate Sprouty. Transcription factors *WT1* and *PPARγ* and *Wnt/β-catenin* signaling pathway have also been shown to upregulate Sprouty. *miR-21* is a cancer-associated microRNA that targets and negatively regulates the Sprouty genes. *TGFβ1* not only downregulates the expression of Sprouty, but also induces the protein degradation via a lysosome-dependent pathway. E3 ubiquitin ligases *c-Cbl*, *Siah2*, *NEDD4*, and *pVHL* induce degradation of Sprouty to regulate its cellular content. *PP2A* competes with c-Cbl for binding to Sprouty, thereby inhibiting c-Cbl-mediated degradation of Sprouty. *Mnk1* is a positive regulator of the Sprouty stability through serine phosphorylation. *c-Cbl* canonical Casitas B-lineage lymphoma; *FZD* receptor Frizzled receptor; *miR-21* microRNA 21; *Mnk1* MAPK-interacting kinase 1; *NEDD4* neural precursor cell expressed, developmentally down-regulated 4; *PP2A* protein phosphatase 2A; *PPARγ* peroxisome proliferator-activated receptor gamma; *RTK* receptor tyrosine kinase; *Siah2* Seven in Absentia homolog 2; *WT1* Wilms tumor suppressor 1; *CS rearrangement* cytoskeletal rearrangement. In this figure, C- and N-terminus of the Sprouty molecule symbol are shown in *white* and *blue*, respectively

Transcriptional and post-translational regulation of the Sprouty cellular content is illustrated in Fig. 1.

### Regulation of the Sprouty activity

Sprouty trafficking to and from the plasma membrane regulates subcellular localization of Sprouty and hence keeps the protein functionality under spatiotemporal control. In unstimulated cells, Sprouty is distributed throughout the cytosol, with hSpry2 being colocalized with microtubules, as well. Upon growth factor activation, Sprouty translocates to the plasma membrane, notably ruffles, where it becomes activated in association with phosphatidylinositol 4,5-bisphosphate (PIP2) and the caveolin-1 (Cav-1) [6, 98–100]. Lim et al. [100] demonstrated that Sprouty binds PIP2 through its CRD domain and that this phenomenon is an essential process for regulation of ERK signaling. As the major structural protein of caveolae (specialized plasma membrane invaginations involved in multiple cellular functions, including signal transduction), Cav-1 similarly inhibits growth factor activation of ERK in a cell density-dependent manner. At higher cell densities, Sprouty/Cav-1 interaction modulates signaling in a growth factor- and Sprouty isoform-specific manner. At lower cell densities, however, Cav-1 inhibits the Sprouty function [101]. Moreover, Hwangpo et al. indicated the interaction between Spry2 and G protein α_o_/G protein-regulated inducer of neurite outgrowth (Gα_o_/GRIN) pathway in modulating Spry2 repression of ERK [102]. Although GRIN was shown to bind and sequester Spry2, the activated Gα_o_ interacted with GRIN to release Spry2.

Sprouty phosphorylation on the conserved tyrosine is considered as an indispensible prerequisite for the regulatory function of Spry1 and Spry2 [99, 103], but not for that of Spry4 [72, 76, 89]. This process, however, is variably induced by different growth factors. Using NIH3T3 fibroblasts transfected with Spry1, Spry2 and Spry4 [89], Mason et al. observed that Spry1, Spry2, but not Spry4 undergo tyrosine phosphorylation after growth factor stimulation. Moreover, although FGF induced tyrosine phosphorylation in both Spry1 and Spry2, PDGF and EGF induced it in Spry1 and Spry2, respectively. Through a time course analysis, they also revealed that FGF-induced tyrosine phosphorylation of Spry1 is kinetically different from that of Spry2. In agreement with earlier reports [87, 103], the investigators found that tyrosine phosphorylation regulates interaction of Sprouty with c-Cbl and concluded that tyrosine phosphorylation serves as a dual feedback loop which, on the one hand, activates Sprouty inhibition of ERK and, on the other hand, promotes c-Cbl-mediated ubiquitination and degradation of Sprouty and thus terminates signaling inhibition. Functional significance of Sprouty phosphorylation on other residues than the conserved tyrosine has also been studied. Rubin et al. [104] observed in HEK293T cells that FGF, but not EGF, activation of ERK is inhibited by Spry2 and that only FGF can induce significant phosphorylation of the C-terminal tyrosines, in particular Tyr227. On this basis, they postulated that C-terminal tyrosine phosphorylation modulates the specificity of the Spry2 inhibition of different ERKs. Results from a study by Aranda et al. supported a functional interaction between dual-specificity tyrosine phosphorylation-regulated kinase 1A (DYRK1A) and Spry2 where DYRK1A regulates the phosphorylation status of Spry2. Since mutation of Thr75 on Spry2, identified as a phosphorylation site for DYRK1A, enhanced the repressive function of Spry2 on FGF-induced ERK signaling, they suggested that DYRK1A is a negative regulator of the Sprouty activity by threonine phosphorylation.

Sprouty dephosphorylation by phosphatases, including protein phosphatase 2A (PP2A) and Src homology-2 containing phosphotyrosine phosphatase (SHP2), can differentially regulate the protein activity. In unstimulated cells, Sprouty is phosphorylated predominantly on serine residues [6]. Lao et al. found that PP2A binds and dephosphorylates Spry2 at Ser112 and Ser115 upon FGF stimulation [105]. They postulated that Spry2 serine dephosphorylation alters the tertiary structure of the protein and thereby exposes the cryptic proline-rich motif in the Spry2 C-terminus, which they had earlier identified as a binding site for Grb2 [106], enabling Spry2 to potently inhibit FGF activation of ERK. They also found that PP2A and c-Cbl compete for binding to Spry2 at an overlapping sequence to fine-tune its activity. They later reported that testicular protein kinase 1 (TESK1) attenuates the ability of Spry2 to inhibit the growth factor actions in a way independent of its kinase activity and primarily by interfering with Spry2/Grb2 interactions and dephosphorylation of serine residues by PP2A [107]. SHP2 has been implicated in regulating the Sprouty activity through dephosphorylation of the phosphotyrosines and subsequent dissociation of Sprouty from Grb2 that positively regulates growth factor activation of ERK [57, 108, 109]. Pan et al. showed that SHP2 regulates Sprouty positively at the transcriptional level and negatively at the post-translational level and concluded that dynamic regulation of Sprouty by SHP2 might be important not only for modulating Ras signaling in developmental processes, but also for RTK signaling in general [37]. On the other hand, Sprouty may mediate its actions in part by increasing active contents of such phosphatases as protein tyrosine phosphatase 1B (PTP1B) and phosphatase and tensin homolog (PTEN). Regulating subcellular localization of PTP1B, Sprouty increases PTP1B soluble content which has been shown to mediate, and mimic, Sprouty-induced repression of cell adhesion and migration [35, 62]. Poppleton et al. reported that Sprouty, regulates cell migration by inhibiting Rac1 activation which they postulated, is mediated in part by PTP1B dephosphorylation of cellular proteins and, subsequently, decreased the amount of phosphorylated p130Cas or phosphatidylinositol 3,4,5-trisphosphate (PIP3) known as Rac1 activators [46]. In a study by Edwin et al. [64], where Spry2 unexpectedly inhibited EGF activation of AKT and exhibited no significant effect on EGF activation of EGFR and ERK, they observed that Spry2 increases the amount and activity of PTEN that was found necessary for Sprouty to attenuate EGF-activated AKT and to inhibit cell proliferation. Beyond its cytoplasmic role in negatively regulating PI3K/AKT, PTEN is phosphorylated and accumulated in the nucleus in response to the Spry2 deficiency to induce p53-mediated growth arrest independently of its phosphatase activity [110]. This process is part of a regulatory mechanism involving Spry2 interaction with PP2A and PTEN for inhibition of tumorigenesis which will be discussed later.

### Regulation of RTK activity and stability

Sprouty might regulate activity and stability of RTKs through interaction with mechanisms involved in reversible (transient) and irreversible (definitive) inhibition of ERK on the basis of dephosphorylation (inactivation) and degradation (downregulation) of RTKs, respectively. Among the reversible inhibitory mechanisms is the RTK dephosphorylation by PTP1B that provides spatiotemporal regulation of the RTK activity [111]. Despite the fact that PTP1B, as described earlier, is regulated by Sprouty to control some cellular functions on the basis of protein tyrosine phosphorylation, there is no evidence of direct interaction between Sprouty and PTP1B in RTK dephosphorylation. In contrast, it is well documented that Sprouty interferes with c-Cbl-mediated downregulation of RTK in a growth factor-dependent manner. Sprouty evidently inhibits c-Cbl-induced ubiquitination and degradation of EGFR, thereby sustaining EGF-activated ERK [7, 87, 103, 112, 113]. Rubin et al. [88] postulated that EGFR activation, followed by Spry2 phosphorylation and its association with c-Cbl, initiates a competitive process where c-Cbl promotes Spry2 polyubiquitination and degradation, and Spry2, conversely, sequesters active c-Cbl molecules and impedes receptor ubiquitination and degradation. They concluded that Sprouty fine-tunes EGF signaling through interlinked positive and negative feedback loops. Moreover, Edwin and Patel [65] suggested a novel role for Sprouty in regulating cellular apoptosis where endogenous Sprouty, by sequestering c-Cbl, augments EGFR activation of ERK and AKT pathways and the resultant anti-apoptotic signaling. A c-Cbl-independent mechanism for Sprouty-induced upregulation of EGFR was identified by Kim et al. [114]. They reported that Spry2 interferes with the trafficking of activated EGFR from early to late endosomes. To do so, Spry2 was postulated to bind the endocytic regulatory protein hepatocyte growth factor-regulated tyrosine kinase substrate (Hrs) to prevent it from interaction with the tumor susceptibility gene 101 protein (Tsg101) which is required for EGFR transport.

### Structural variation and functional divergence of the Sprouty proteins

The C-terminal cysteine-rich (CRD) domain of Sprouty is a highly conserved region implicated for such key functions of the protein as a membrane translocation and ERK inhibition [61, 87, 98, ]. The N-terminal tyrosines Tyr53 (in Spry1 and Spry4) and Tyr55 (in Spry2) are also conserved residues crucial for the Sprouty functionality to the extent that their corresponding dominant-negative mutants fail to attenuate ERK signaling and even repress the function of the wild type (WT) protein [60, 72, 76, 89, 99]. Less homologous sequences, however, have been localized in the C-terminal as well as in the N-terminal regions of the Sprouty isoforms that contribute to their differential interaction with signaling molecules and molecular partners and accounts, in part, for their functional divergence. Sprouty isoforms display differential affinity for different molecular targets upstream or downstream of Ras or even beyond ERK. Although interacting with Raf kinases [115–118], Spry2 exhibits the highest affinity for Grb2. Lao et al. identified an exclusive proline-rich sequence in the Spry2 C-terminus which was found as a binding site for Grb2 responsible for differential interaction of Spry2, as compared to Spry1 and Spry4, with Grb2 [106]. Sasaki et al. [76] demonstrated that the Spry4 mutants lacking the N-terminal conserved tyrosine residue necessary for suppressing FGF signaling still inhibit the VEGF-A-induced activation of ERK in a Ras-independent manner by binding through the CRD domain to Raf1, indicating that Spry4 differentially regulates different ERK pathways through distinct action points. Later, in a study by Ayada et al. [119], the CRD domain was further implicated for the Spry4 functions in regulating the VEGF-A-induced, protein kinase C- (PKC-) dependent activation of ERK, as well as various types of PLC-dependent signaling. The investigators indicated that Spry4 interacts through its CRD domain with PIP2 to inhibit PIP2 hydrolysis and ensuing activation of PKC in response to VEGF-A. Also found to impact the PKC downstream signals, Spry4 was introduced as a general inhibitor of phospholipase C (PLC) and PLC-dependent signaling with regulatory functions broader than previously thought [119]. In a parallel study, investigating the physiological function of Spry4 as an angiogenic regulator [120], they indicated that Spry4 suppresses Ras-independent angiogenesis stimulated by VEGF-A and sphingosine-1-phosphate (S1P) while it does not affect Ras-dependent VEGF-C signaling. TESK1, a cofilin kinase with critical role in integrin-mediated actin cytoskeletal reorganization and cell spreading, was identified by Tsumura et al. [49] as a target for the Spry4 CRD domain through which Spry4 binds TESK1 and inhibits cofilin phosphorylation, thereby negatively regulating cell spreading and migration independently of its regulatory effect on ERK.

Variation in the binding sites for such molecular partners as c-Cbl, CIN85, and Cav-1 is also documented. Known to mediate monoubiquitination of activated RTKs [121–123], c-Cbl interacts with endocytic scaffold complexes, including CIN85/endophilins, to facilitate RTK endocytosis and degradation [124–126]. The N-terminal c-Cbl binding motif is shared by Sprouty isoforms. However, Spry2 exhibits the highest binding affinity for c-Cbl and Spry4 weakly binds it [113, 127]. Mason et al. [89] found that tyrosine phosphorylation is essential for Sprouty association with c-Cbl and that a less homologous sequence within the c-Cbl binding motif of Spry4 prevents it from phosphorylation and binding to c-Cbl. Furthermore, principal CIN85-binding sites are found only in Spry1 and Spry2. Haglund et al. showed that Spry2 associates with c-Cbl and CIN85 upon EGF stimulation to inhibit EGFR endocytosis and degradation, whereas Spry4 fails to inhibit downregulation of EGFR [128]. Sprouty isoforms have also shown differential interaction with Cav-1. Cabrita et al. showed that although all four Sprouty isoforms can bind Cav-1 through their conserved C-terminal domain, they exhibit differential cooperativity with Cav-1 in repressing ERK [101]. When either Spry1 or Spry3 were expressed in the presence of Cav-1, FGF-induced ERK activation was synergistically attenuated. However, when either Spry2 or Spry4 were present along with Cav-1, ERK activation increased slightly compared with when Cav-1 was present by itself, suggesting a decrease in the inhibitory activity of Cav-1. In addition, it was shown in another study that inhibitory function of the Sprouty proteins is enhanced through cooperative interaction among the protein isoforms. Ozaki et al. [129] found that all four Sprouty isoforms are able to form hetero- and homo-oligomers through their C-terminal domains. They observed that while Spry1 and Spry4 interact with Grb2 and Sos1, respectively, the hetero-oligomer formed by the two exhibits the most potent inhibitory effect on FGF-activated ERK.

## Deregulation of Sprouty in cancer

Given their critical role as modulators of MAPK/ERK and mediators of the crosstalk between ERK and other signaling pathways for maintaining homeostatic control of cellular behavior, the Sprouty proteins are conceivably expected to be deregulated in malignant conditions. On this basis, deregulation of Sprouty in a variety of cancers has been studied by different investigators and its utility as a biological marker [21, 69, 110, 130–134 ], a tumor suppressor [41, 52, 66, 135–140], or even an oncogene [55, 68–70, 141, 142] with application in targeted approaches [13, 52, 56, 69, 131, 134, 143, 144] has been argued which are discussed below.

### Breast cancer

In a study by Lo et al. in 2004, although mSpry1 and mSpry2 were implicated in the breast development during puberty and pregnancy [21], it was revealed in Cancer Profiling Array containing pairs of complementary DNAs (cDNAs) generated from 50 matched pairs of normal and cancer tissues that *hSpry1* and *hSpry2* were consistently downregulated in breast cancer. Real-time PCR confirmed that more than 90 % of the patient samples demonstrated suppressed expression of Spry1 and Spry2. Neither DNA methylation nor histone hypoacetylation was found to be responsible for the *Sprouty* downregulation by an epigenetic silencing. They finally indicated that the MCF-7 breast cancer cells transfected with a dominant-negative mutant of Spry2 proliferated faster and exhibited anchorage-independent growth *in vitro* and formed larger tumors *in vivo*. Following a meta-analysis of the gene expression profiles of a total of 1,107 tumors combined with a further analysis of two single datasets, Faratian et al. [132] reported in 2011 that *Spry1*, *Spry2*, and *Spry4* were differentially expressed across clinicopathological subgroups of the breast cancer and that low *Spry2* expression was associated with high expression of the human epidermal growth factor receptor 2 gene *(HER2)*. Spry2 was found as an independent prognostic factor that may identify breast cancer patients with a more favorable outcome even when tumors exhibit poor pathological features. Since Spry2 was shown to act synergistically with the HRE2-targeting trastuzumab to reduce cell viability *in vitro*, the expression of Spry2 was quantified in a cohort of 122 trastuzumab-treated patients, revealing that low Spry2 expression was associated with poor outcome and increased risk of death. Hence, the investigators argued for the usefulness of Spry2 in stratifying patients for treatment with trastuzumab.

Implicating the urokinase-type plasminogen activator receptor (uPAR) as a partner protein interacting with hSpry1 [145], Mekkawy et al. reported that hSpry1 colocalizes with uPAR upon stimulation with EGF and urokinase-type plasminogen activator (uPA), and suppresses uPAR-mediated migration and invasion of the MDA-MB-231 breast cancer cells [59]. Vanas et al. [146] recently indicated that different breast cancer cell lines differentially express Sprouty as compared with normal mammary epithelial cells. However, a correlation between the expression profiles of *Spry2* and *Spry4* was found. They also reported that ectopic expression of Spry4 inhibited cell proliferation independently of its endogenous expression level. Furthermore, increased Spry4 interfered with serum-induced activation of ERK and inhibited cell migration.

### Prostate cancer

In 2004, Kwabi-Adoo et al. [143] reported the result of immunohistochemical analysis of 407 tissue microarrays containing prostate cancer, as well as matched normal tissue cores, showing downregulation of hSpry1 in approximately 40 % of the prostate cancer cases studied. This finding was corroborated by real-time PCR where Spry1 mRNA levels were significantly decreased in 16 out of 20 prostate cancer tissue samples in comparison with the normal tissue. In their *in vitro* study, the investigators interestingly observed that the prostate cancer cells LNCaP and PC3, in contrast to primary epithelial cells, did not show induction of the Spry1 expression at mRNA and protein levels in response to FGF2 stimulation. They also reported that Spry1 transfection of LNCaP and PC3 cells had an inhibitory effect on colony formation and cell proliferation. In agreement with earlier studies showing upregulation of FGFs in prostate cancer, Kwabi-Adoo et al. concluded that Spry1 downregulation may lead to the unrestrained FGF-induced signal transduction and hence tumor progression. Later, McKie et al. [135] observed that Spry2 mRNA is downregulated in invasive prostate cancer cell lines as well as in clinically high-grade prostate cancers when compared to benign prostatic hyperplasia (BPH) and well-differentiated prostate tumors. Identifying hypermethylated CpG islands in *hSpry2* gene correlated with suppressed expression of hSpry2 mRNA, they implicated epigenetic inactivation as the main mechanism for the *hSpry2* downregulation in prostate cancer. As later reported by Kwabi-Adoo et al., this mechanism is also responsible for downregulation of *Spry1* in prostate cancer [147]. Data from an integrated genomic profiling of 218 prostate tumors by Taylor et al. revealed that *Spry1* and *Spry2* genes are inactivated in 15 and 18 % of the primary cancer, as well as in 42 and 74 % of the metastatic disease, respectively [148]. Through *in situ* hybridization on 14 prostate tissue samples and quantitative real-time PCR analysis in 25 pairs of matched normal and tumor tissue samples, downregulation of Spry4 in a subset of prostate cancers was reported by Wang et al. [51]. Their epigenetic analysis revealed methylation of a CpG island in the 5′-regulatory region of *Spry4* in more than a half of all prostate cancer DNA samples studied which was significantly correlated with decreased expression of Spry4 mRNA. They also demonstrated that Spry4, unlike Spry1, does not hinder cell growth but rather inhibits cell migration, suggesting that Spry1 and Spry4 perform different functions in prostate cancer. Later, Fritzsche et al. [149] observed through microarray analysis of microdissected prostate tissue specimens a coordinated, yet modest, downregulation of both Spry1 and Spry2 mRNAs gradually increasing from hyperplasia to severe prostatic intraepithelial neoplasia (PIN) to cancer. Spry2 mRNA downregulation was confirmed in an independent, larger series of macrodissected tumors by quantitative RT-PCR. Unlike McKie et al., however, they reported that *Spry2* downregulation in prostate cancer is independent of DNA methylation.

ERK and PI3K/AKT have been identified as the two most commonly altered pathways in prostate cancer with alteration frequency of 42–43 % in primary and 90–100 % in metastatic prostate cancer [148]. Activation of ERK and PI3K/AKT by aberrant RTK signaling has been implicated in the development of aggressive prostate cancer [150]. On this basis, Sprouty interactions with other feedback regulators of the two pathways and its significance in prostate cancer tumorigenesis and progression have been explored by some investigators. *PTEN* has been reported to be inactivated in 4 % of the primary prostate cancer as well as in 42 % of the metastatic disease [148]. A key genetic interaction between the Sprouty and PTEN genes has been reported. Although *Pten* heterozygosity *per se* results in low-grade PIN in mice [151], Schutzman et al. [140] showed that concomitant inactivation of the Sprouty genes (*Spry1* and *Spry2*) accelerated emergence of PIN and promoted development of more extensive, high-grade phenotype along with the transition to invasive cancer. Conversely, expression of a *Spry2* gain-of-function transgene in the context of *Pten* homozygosity suppressed the AKT hyperactivation, and the prostate tumorigenesis resulted from *Pten* loss-of-function, implicating the Sprouty genes in regulation of ERK and PI3K/AKT pathways in prostate cancer. They suggested that the expression status of the PTEN and Sprouty genes in prostate biopsies from men at risk for prostate cancer could potentially help to risk-stratify patients with PIN. Patel et al. later indicated that Sprouty status along with that of PTEN and PP2A collectively represents an important determinant of the prostate cancer progression [110]. They showed in a coherent set of *in vitro* and *in vivo* systems that although Spry2 deficiency is sufficient to activate both PI3K/AKT and ERK cascades, it is insufficient to drive tumorigenesis with Spry2-deficient cells exhibiting PTEN-mediated growth arrest. As follows, it was shown that the Spry2 deficiency-induced growth arrest mechanistically involves PTEN, PP2A, glycogen synthase kinase 3 beta (GSK3B), p53, and reactive oxygen species (ROS). By enhancing RTK activation, Spry2 deficiency increases intracellular ROS which subsequently activates PP2A. PP2A then dephosphorylates and activates GSK3B that drives phosphorylation and nuclear accumulation of PTEN. Nuclear PTEN eventually promotes growth arrest by induction of p53 and p21, independent of its phosphatase activity. Overall, by introducing a novel, PP2A-dependent tumor suppressor checkpoint, Patel et al. identified the cooperative role of concomitantly inactivated Spry2, PTEN, and PP2A to drive the prostate cancer progression. Hence, they postulated that loss of Spry2 may represent an early event in prostate carcinogenesis compensated by nuclear PTEN-mediated growth arrest which might be subsequently overcome by inactivation of PTEN, TP53, or PP2A.

### Liver cancer

In a gene expression study by Chen et al. in 2002, *Spry2* was among the top 600 genes found to be differentially expressed in hepatocellular carcinoma (HCC) compared with non-tumor liver tissue [152]. In 2006, a more stringent and biologically relevant analytic approach to the same database by Fong et al. revealed a consistent downregulation of *Spry2* in HCC [50]. Using *in situ* hybridization on tissue microarrays from an independent set of patients, they confirmed significantly differential expression of Spry2 in HCC compared with normal or cirrhotic liver tissue. Although showing the resemblance between the expression pattern of Spry2 and that of several potential tumor markers in hepatocellular carcinoma, the investigators ruled out loss of heterozygosity (LOH) or the promoter hypermethylation as possible mechanisms responsible for *Spry2* downregulation. Moreover, it was shown that Spry2 plays functionally important roles in HCC by inhibiting hepatocyte growth factor (HGF)-induced cell proliferation and ERK activation in the Spry2-overexpressing HCC cells.

Identifying *Spry2* in their genomic analysis as a downregulated and frequently deleted gene in HCC, Lee et al. [66] observed *in vitro* that overexpressed Spry2 inhibits HCC cell growth. Their *in vivo* study using hydrodynamic transfection not only exhibited, in line with earlier studies [153, 154], the cooperative role of activated Wnt/β-catenin and Ras in induction of HCC, but also revealed that dominant negative Spry2 cooperates with β-catenin to induce development of liver cancer in mice, with tumor cells showing upregulation of ERK and deregulation of genes involving in cell proliferation, apoptosis, and angiogenesis. This study suggested that Spry2 might function as a tumor suppressor in HCC. They reported later the synergistic role of Spry2 inactivation and c-Met upregulation in mouse and human hepatocarcinogenesis [155]. They observed in a collection of human liver tissue samples the significant downregulation of Spry2 protein as well as ubiquitously high expression of c-Met (total and activated) and its downstream effectors (activated Erk and Akt) in most cases of HCC with poorer outcome (HCCP) in the context of WT Ras. The expression of Spry2 was found to be downregulated at transcriptional and post-translational levels by promoter hypermethylation, LOH, and proteasomal degradation by NEDD4. *In vitro*, Spry2 overexpression inhibited c-Met-induced cell proliferation as well as ERK and AKT activation, whereas loss of Spry2 potentiated c-Met signaling. Their *in vivo* study with mice hydrodynamically transfected with c-Met and/or a dominant negative mutant form of Spry2 indicated that Spry2 inactivation cooperates with c-Met to induce hepatocarcinogenesis by sustaining proliferation and angiogenesis, suggesting a pivotal oncogenic mechanism responsible for unrestrained activation of ERK and AKT pathways in human hepatocarcinogenesis. By hydrodynamic injection and coexpression of an activated/myristoylated form of Akt (myr-Akt) and a dominant negative Spry2 mutant in the mouse liver, Wang et al. [71] later indicated that loss of Spry2 accelerated AKT-induced hepatocarcinogenesis which was associated with activation of ERK pathway and pyruvate kinase M2 (PKM2)-induced glycolysis. *In vitro*, they found that activation of PKM2 in the HCC cell line HLE transfected with Akt and dominant negative Spry2 is independent of ERK and AKT cascades, collectively implying that loss of Spry2 synergizes with activated AKT to induce rapid hepatocarcinogenesis through the activation of ERK and PKM2 pathways.

Differential expression of the Sprouty homologs in HCC was reported by Sirivatanauksorn et al. [133], where paired HCC and non-tumor liver tissue samples from 31 patients were examined by quantitative RT-PCR. Most HCC tissues showed upregulation of *Spry1* and downregulation of *Spry2* and *Spry4* at mRNA level. Moreover, mRNA expression of *Spry1*, *Spry2*, and *Spry4* in cancerous specimens was significantly different from that in nontumor tissues. The expression of *Spry3*, however, did not show any significant difference among the samples. Studying the association of the Sprouty gene expression with clinical parameters of HCC, they indicated that the expression of *Spry2* was significantly lower in patients with advanced disease and angiolymphatic invasion, whereas *Spry1* was significantly upregulated in cases without underlying cirrhosis compared with cirrhotic patients. Prognostic significance and clinical relevance of the Spry2 protein expression in HCC was later studied by Song et al. [134]. Their initial study *in vitro* showed that the ratio of phospho-ERK to Spry2 in the HCC cell lines MHCC97L, HCCLM3, and HCCLM6 displayed an elevation concordant with their stepwise metastatic potential. Similarly, the Spry2 expression *per se* inversely correlated with the metastatic potential of the HCC cells. In their immunohistochemical study, they found that 86.3 % of a total of 240 patients exhibited Spry2 downregulation. They reported that Spry2 downregulation accompanied highly malignant clinicopathological features like advanced TNM stages and tumors with vascular invasion and poor differentiation. They found that Spry2-negative patients had poorer survival and increased postoperative recurrence, and thereby suggested potential implications of Spry2 as a predictor of the disease prognosis and a biomarker of the treatment sensitivity.

### Lung cancer

Using reverse transcription-PCR and immunohistochemical staining of matched tumor and nontumor samples, Sutterluty et al. reported in 2007 that *Spry2* expression, but not that of *Spry1*, is consistently reduced at mRNA and protein levels in non-small cell lung cancer (NSCLC) tissues [52]. Their *in vitro* analysis with a panel of NSCLC cell lines revealed that high levels of Spry2 expression were exclusively detected in KRAS-mutated cells and that only few cell lines with reduced Spry2 exhibited Spry2 promoter hypermethylation. Moreover, although ectopic expression of Spry2 inhibited ERK activity and diminished cell migration in NSCLC cells with WT KRAS, but not in those with the mutated one, it significantly reduced cell proliferation in all NSCLC cell lines studied *in vitro* and blocked tumor formation in mice inoculated with the KRAS-mutated cell line A-549. In addition, even a dominant negative Spry2 mutant defective in antagonizing ERK significantly, although less potently, inhibited cell proliferation in NSCLC cells with or without KRAS mutation. Collectively, they demonstrated that Spry2 downregulation contributes to NSCLC tumorigenesis via ERK-dependent and ERK-independent mechanisms and implicated Spry2 as a tumor suppressor in NSCLC. Consistently, Shaw et al. reported that Spry2 functions as a tumor suppressor in the context of a germline oncogenic KRAS mutation—KRAS^G12D^—in which loss of Spry2 increased the number and overall burden of lung tumors in mice [31]. This was corroborated by a later report whereby lack of Spry2 expression along with high level of ERK activation was evident in putative tumorigenic cells of KRAS^G12D^-induced neoplasia in mouse lungs [156]. The role of Spry2 in inhibiting lung tumor development was further confirmed by Minowada et al. [144] who evaluated consequences of Spry2 overexpression in mouse lung epithelium in the context of urethane-induced tumorigenesis. The chemical carcinogen urethane induces KRAS gain-of-function mutations and lung tumors in mice. The investigators observed that Spry2 overexpressing animals developed significantly fewer and smaller tumors compared with their littermate controls. Since the overexpression of Spry2 did not alter KRAS mutational frequencies, it was suggested that the tumor-suppressing activity of the overexpressed Spry2 might be applied at stages of carcinogenesis subsequent to KRAS mutation.

A putative role for Spry4 as part of Wnt7A/Fzd9 tumor-suppressing pathway was initially suggested by Winn et al., where Wnt7A and Fzd9 induced the expression of Spry4 in NSCLC cells [136]. They subsequently identified PPARγ [157, 158] and Spry4 [56] as downstream effectors of Wnt7A/Fzd9 that mediate its anti-tumorigenic effects. Reporting downregulation of Spry4 in a variety of NSCLCs as well as in dysplastic lung cell lines, Tennis et al. showed that Spry4 transfection inhibited NSCLC cell growth, migration, invasion, and epithelial-mesenchymal transition. They found that Wnt7A/Fzd9 signaling increases Spry4 promoter activity through PPARγ. Corroborated by their earlier reports [158, 157, 136], Tennis et al. concluded that Spry4 represents an inducible effector of the Wnt7A/Fzd9 pathway downstream of PPARγ which restores a nontransformed epithelial phenotype while inhibiting NSCLC cell growth, migration, and invasion [56].

### Colon cancer

In their cDNA array study on different cancers in 2004, Lo et al. presented differential expression pattern of *Spry2* in 38 matched pairs of normal and tumor samples from colon cancer patients [21]. In a BLAST search of human ESTs followed by *in silico* expression analysis, Katoh and Katoh [13] observed that *Spry4* mRNA is expressed in colon cancer. Based on the comparative genomics analyses, they characterized *Spry4* as the evolutionarily conserved target gene of the Wnt/β-catenin signaling pathway. Implicating Spry4 in Wnt/β-catenin regulation of progenitor cells, they suggested that epigenetic silencing and loss-of-function mutations of *Spry4* could lead to carcinogenesis. In a study by Barbachano et al. [69], immunofluorescence analysis of human colon cancer biopsies quantitatively confirmed in 34 patients showed high levels of Spry2 and low levels of E-cadherin in undifferentiated, high-grade tumors in contrast to low levels of Spry2, and high levels of E-cadherin in low-grade specimens. *In vitro*, Spry2 and E-cadherin exhibited an inverse correlation and reciprocal regulation in colon cancer cells. The investigators found that Spry2 induces the expression of the ZEB1 epithelial-to-mesenchymal transition gene and protein and abrogates the induction of an adhesive epithelial phenotype by 1,25(OH)2D3. Supplemented by a meta-analysis of the data available at the Oncomine database [159] in favor of higher expression of *Spry2* in colon tumors compared with other neoplasias, their results suggested a tumorigenic action and a potential role as a tumor marker for Spry2 in colon cancer. Examining a low number of matched colon cancer samples, Holgren et al. [68] also reported upregulation of Spry2, as well as c-Met, at mRNA and protein levels. *In vitro*, Spry2 upregulation in the KRAS-mutated cell line HCT-116 significantly increased cell proliferation, accelerated cell cycle transition and enhanced cell migration and invasion which were attributed, at least in part, to activation of HGF/c-Met axis and its downstream effectors Akt and Erk. They also demonstrated that Spry-2 knockdown significantly inhibited cell invasion in both WT- and mutant KRAS-expressing cell lines. With Spry2 transfectants forming significantly larger xenografts with higher metastatic potential *in vivo*, they concluded that Spry2 may control metastatic potential of colon cancer cells, at least in part, by c-Met upregulation. Examining primary tumor samples from 113 patients with colorectal cancer, Watanabe et al. [160] later observed that KRAS mutant tumors (31 %) exhibited a distinct gene expression signature compared with their WT counterparts (69 %) where Spry2 was among the 30 genes upregulated in the KRAS mutants. They found that the discriminating genes identified were related to not only K-Ras/ERK but other signaling pathways such as Wnt/β-catenin, NF-kappa B activation, and TGFβ signaling, thereby suggesting a crosstalk between K-Ras-mediated signaling and other pathways in colorectal cancer.

In contrast, Feng et al. reported in 2010 that Spry2 may be a potential biomarker in predicting the response to anti-EGFR treatment in colon cancer [131]. They showed that the expression of Spry2 positively correlates with the sensitivity of colon cancer cells to the EGFR inhibitor gefitinib and that Spry2 can enhance the response of colon cancer cells to gefitinib by increasing the expression of phosphorylated EGFR, total EGFR, and PTEN. They later reported downregulation of Spry2 in association with colon cancer progression and suggested a tumor suppressor role for Spry2 [137]. By real-time quantitative RT-PCR on mRNA isolated from normal and tumor tissues of 67 patients with colon cancer, they showed that *Spry2* was downregulated in 72.7 % (16/22) of stage II, 91.3 % (21/23) of stage III, and 100 % (22/22) of stage IV tumors examined. A negative correlation was also evident between the expression levels of Spry2 and the microRNA miR-21, an indicator of poor survival and poor response to adjuvant chemotherapy in cancer patients. *In vitro*, overexpressed Spry2 inhibited the growth and migration of HCT116 human colon cancer cells which was concomitantly accompanied by an increase in the expression of PTEN and reduction in phosphorylation of ERK and Akt. Spry2 also suppressed the growth and tumorigenesis of colon cancer cells *in vivo*. In line with earlier studies suggesting Sprouty genes as targets of miR-21 [161–163], they proposed that Spry2 is negatively regulated by miR-21 and that such interaction may play a role in colon cancer carcinogenesis. Spry1, too, was later found to inhibit EGF- or uPA-induced migration of HCT116 cells *in vitro* [59].

### Melanoma

Examining a panel of melanocytic and melanoma cell lines, Tsavachidou et al. reported in 2004 that Spry2 acts as an inhibitor of ERK signaling in melanocytes and WT BRAF melanoma cells, but not in cell lines with BRAF^V600E^ (previously designated as BRAF^V599E^) mutation [117]. Their genetic and gene expression analyses revealed that Spry2 is downregulated in melanoma cells harboring WT BRAF yet upregulated in the BRAF^V600E^ mutants. They observed that Spry2 directly interacted with WT B-Raf and inhibited ERK but failed to directly bind the mutant B-Raf and did not affect ERK. In conclusion, they proposed that Spry2 may be bypassed in melanoma cells either by downregulation of its expression in WT BRAF cells or through BRAF mutation. In a later study [164], microarray data validated by real-time PCR indicated upregulation of *Spry2* in melanoma cell lines with mutations in BRAF and NRAS. Qi et al. [95] showed that the expression of a dominant-negative Siah2 RING finger mutant in SW1 mouse melanoma cells reduced their tumorigenesis through the increase of Spry2. Using genomic and gene expression analyses of an animal model of skin neoplasm that produces both benign and malignant tumors, Quigley et al. [165] demonstrated that alleles that are not relevant in normal tissue are associated with tumor susceptibility but somatic alterations during tumor progression may reduce the detectable influence of germline polymorphisms. As such, although *Spry2* was identified as a susceptibility gene for skin tumors and was expressed at very low levels in normal skin and at elevated levels in tumors, higher *Spry2* expression in tumors was found to be associated with greater resistance to tumorigenesis which was ascribed to the role of Spry2 in regulation of ERK. Through an integrative approach analyzing genomic and gene expression changes in relation to *in vivo* growth aggressiveness, Mathieu et al. [166] found that genomic loss of *Spry1* and *Spry2*—along with altered expression of some other genes—was associated with a more aggressive melanoma phenotype. However, no convincingly enhanced levels of ERK phosphorylation were found in the fast-growing subgroup of their melanoma model compared with its slow-growing counterpart. Given widespread activating mutations in BRAF and, in particular, inability of Spry2 to attenuate ERK in the context of BRAF^V600E^ mutation, their findings argue for a role of Sprouty in regulation of melanoma aggressiveness independent of attenuation of ERK.

### Sarcoma

In 2005, gene expression profiling of 134 human sarcoma tumors by Baird et al. revealed upregulation of *Spry2* in 2/7 of fibrosarcomas and 4/5 of dermatofibrosarcomas [167]. Lito et al. reported upregulation of Spry2 protein in the human fibrosarcoma cell lines SHAC, HT1080, VIP:FT and NCI as well as in HRAS- and NRAS-transformed human fibroblasts [141]. They provided evidence that Spry2 is necessary for sarcoma formation by patient-derived fibrosarcoma cell lines or HRAS oncogene-transformed human fibroblasts through EGFR signaling. Indicating Spry2-dependent interaction of H-Ras with c-Cbl and CIN85, and the ability of Spry2 to sustain EGFR signaling and tumor formation in the context of HRAS activation, they indicated that the positive effect of Spry2 in sarcoma tumor formation by human fibroblasts is specific to HRAS transformation. Contrasting the role of Spry2 in HRAS transformation with that earlier reported in KRAS^G12D^ mutation [31], Lito et al. raised the possibility of the differences among oncogenic Ras isoforms in regulation of tumorigenesi resulted from Ras isoform-specific modes of action [141]. Using a similar model system, they later showed that oncogenic HRAS requires Spry2 to protect fibroblasts from UV-induced apoptosis and damage and to resist cisplatin cytotoxicity. This antiapoptotic function of Spry2 was found to be mediated by a pathway consisting of Akt, human double minute 2 (HDM2), and p53 recruited through Rac1 [55]. In another study, gene expression profiling by Schaaf et al. [70] revealed that *Spry1* and *Spry2*, and *Spry4* were consistently upregulated in the embryonic subtype of rhabdomyosarcoma (ERMS) as compared with its alveolar subtype (ARMS). They indicated that elevated Spry1 in ERMS cells associated with hyperactive ERK signaling is caused by oncogenic RAS mutations which is frequent in ERMS but absent in ARMS. Spry1 was found essential for ERMS cell proliferation and survival *in vitro* and ERMS tumor formation and maintenance *in vivo*. Accordingly, silencing of Spry1 abolished tumorigenicity of ERMS cells and caused regression of established ERMS tumors in mice. Thus, they argued that Spry1 functions as an agonist of ERK signaling in rhabdomyosarcoma with RAS mutation.

A microarray analysis of 41 soft tissue tumors reported by Nielsen et al. in 2002 [168] revealed that *Spry1*, *Spry4*, and *KIT* were among the genes that demonstrated specific expression in gastrointestinal stromal tumors (GISTs). Using expression profiling of the GIST882 cells treated with the c-Kit inhibitor imatinib *in vitro*, Frolov et al. later identified *Spry4* as an imatinib-responsive gene significantly downregulated in the treated cells and the Spry4 protein as a downstream effector of the c-Kit-activated ERK targeted by the drug [130]. In their clinical study, since Spry4 levels were dramatically decreased in patients responsive to the drug compared with non-responsive patients, the authors proposed Spry4 as a reliable marker of the imatinib-responsive treatment.

Sprouty, on the other hand, has reportedly shown inhibitory effects on other types of sarcoma cells and tumors. Identifying Spry2 as an inducible, negative regulator of HGF/SF-induced activation of ERK and AKT, Lee et al. [63] reported in 2004 that Spry2 inhibits proliferation, anchorage-independent growth, migration, and invasion of SK-LMS-1 human leiomyosarcoma cells *in vitro*. Rathmanner et al. reported that Spry2, but not Spry4, potently inhibits proliferation and interfere with migration of human osteosarcoma-derived cells, with the N-terminal sequence variation being implicated in the specific inhibitory effect of Spry2 [169]. Osteosarcoma cell invasion was also shown to be impeded by overexpressed Spry1 as a result of interaction with uPAR [59]. A microarray study by Holtkamp et al. [170] identified *Spry2* as one of the genes differentially upregulated in benign human neurofibroma as compared with malignant peripheral nerve sheath tumors (MPNST) from the same patient. Supporting a role for Sprouty in limiting the development of these benign lesions, Courtois-Cox et al. later reported that Sprouty genes were highly expressed in both Raf-expressing and neurofibromin 1 (NF1)-deficient fibroblasts [171]. They argued that Sprouty is part of a multifaceted negative feedback signaling network in response to the aberrant activation of Ras that underlies oncogene-induced senescence.

### B-cell lymphoma

Reported in 2008, epigenetic silencing of *hSpry2* and its clinical relevance in lymphoid/hematopoietic malignancies were investigated by Sanchez et al. [172]. Of 16 relevant human cancer cell lines, *hSpry2* promoter was methylated only in the B-cell diffuse lymphoma cell line HT. This was found to be associated with and related to *hSpry2* downregulation at mRNA and protein levels. The ectopic expression of hSpry2 in HT cells drastically reduced the phorbol 12-myristate-13-acetate (PMA)-induced activation of ERK. The investigators then observed that HT mock cells developed tumors in nude mice seven times larger than those formed by the hSpry2 transfectants. Clinically, they identified hSpry2 hypermethylation in 26 out of 71 patients with B-cell diffuse lymphoma as well as in 10 out of 13 Burkitt’s lymphomas but in no normal B lymphocytes from 37 healthy individuals. As evaluated in 55 out of the initial 71 patients, the authors reported that Spry2 promoter hypermethylation was significantly associated with a lower 5-year survival rate and concluded that Spry2 could be an important regulator in mouse B-cell diffuse lymphomas. In agreement, epigenetic silencing and repressed expression of *Spry2* in mouse and human mature B-cell tumor cell lines and a *T-cell leukemia 1*-transgenic (*TCL1*-tg) mouse model of B-cell lymphoma as well as in human B-cell lymphoma samples were reported by Frank et al. [67]. Five out of seven diffuse large B-cell lymphomas and the only Burkitt’s lymphoma sample studied contained DNA methylation of the *Spry2* promoter which was associated with repressed *Spry2* expression in 4 out of 6 lymphoma samples. Mechanistically, they demonstrated that Spry2 overexpression reduces ERK activation and induces B-cell apoptosis and Spry2 inactivation, on the other hand, increases ERK-dependent proliferation of B-cells. In conclusion, they implicated Spry2 in regulation of TCL1-augmented ERK signaling and B-cell proliferation and suggested Spry2 epigenetic silencing as an aberration contributing to B-cell lymphoma progression.

### Testicular germ cell cancer

Results from a genome-wide scan among 277 cases of testicular germ cell tumors (TGCT) and a subsequent replication study on 371 cases were reported by Kanetsky et al. in 2009 [142] whereby genetic variation of *KITLG* (gene encoding the ligand for the receptor tyrosine kinase c-KIT) and *Spry4* was shown to predispose to testicular germ cell cancer. These findings were found in agreement with an earlier report identifying Spry4 as downstream of c-KIT activation of ERK which is upregulated in GISTs in association with aberrant activation of c-KIT [130].

### Endometrial cancer

Differential expression of Spry2 in normal endometrium throughout the menstrual cycle as well as in endometrial cancer was reported by Velasco et al. [41] in 2011. Indicating a complete absence of Spry2 in about 20 % of 136 cases immunohistochemically studied, they found that Stage III and IV tumors had the lowest levels of Spry2 immunostaining. Moreover, a strong, inverse correlation between the Spry2 expression and the cell proliferation index Ki67 was revealed, with nonendometrioid carcinomas (NEEC) exhibiting the highest level of cell proliferation and lowest level of the Spry2 expression. They concluded that Spry2 may be involved in regulation of endometrial carcinogenesis through control of cell proliferation.

### Thyroid cancer

In 2012, Macia et al. [139] reported that Spry1 is expressed in mouse thyroid C-cells and that targeted deletion of *Spry1* causes C-cell hyperplasia, a precancerous lesion preceding medullary thyroid carcinoma (MTC), in young adult mice. They also found that ectopic expression of Spry1 in a tumorigenic, MTC-derived cell line reduced proliferation of the cancer cells *in vitro* and inhibited growth of the xenografts *in vivo*. Furthermore, they indicated that the *Spry1* promoter is frequently methylated and that the *Spry1* expression is accordingly decreased in human MTC samples, collectively suggesting that Spry1 is a candidate tumor-suppressor gene in MTC. By *in vivo* analysis of the thyroid glands from the Spry1 knockout mice, they recently described a novel mechanism by which Spry1 induces a senescence-associated secretory phenotype via activation of the NF-kappaB pathway, thereby restricting cell proliferation independently of the ERK pathway [173].

### Pituitary tumor

Investigating the role of the C-terminal binding protein (CtBP), a transcriptional corepressor with known oncogenic properties, in normal and neoplastic pituitary, Dorman et al. [138] identified Spry2 as a potential target of CtBP1 and hence a potential tumor suppressor involved in regulation of pituitary cell growth and apoptosis. Gene expression profiling validated by real-time PCR and Western blotting revealed that Spry2 is upregulated in the CtBP1-deficient GH4 pituitary tumor cells that grow slower than their parental cells. Mechanistically, upregulation of Spry2 in CtBP1-deficient GH4 cell was shown to impair phosphorylation of the fibroblast growth factor receptor substrate 2 (FRS2α) in response to FGF.

### Ovarian cancer

In a study by Polytarchou et al. [174], Spry1 was identified as a target of miR-21 in Akt2-conferred resistance to hypoxia in both normal and tumor cells. Upon oxygen deprivation, Akt2 was found to induce miR-21 which in turn targets and downregulates *Spry1*, *PTEN*, and *programmed cell death 4 (PDCD4)* led to enhanced survival of Akt2-expressing cells during hypoxia. They provided evidence that this hypoxia-activated, Akt2-dependent pathway is present in ovarian cancer through examining a panel of ovarian cancer cell lines *in vitro* as well as real-time PCR analysis of 31 human ovarian cancer samples. In their initial report of an ongoing investigation into the role of Sprouty in ovarian cancer, Masoumi-Moghaddam et al. later documented differential expression of Spry1 and Spry2 in a panel of ovarian cancer cells where a tendency for downregulation of Spry1 and/or Spry2 was evident [175].

### Clear cell renal cell carcinomas (ccRCC)

In an attempt to identify genes effectively discriminating between clinically aggressive and nonaggressive ccRCC, Takahashi et al. performed the gene expression profiling of 29 tumors obtained from patients with diverse clinical outcomes. According to their report, *Spry1* was exclusively upregulated in the good outcome group [176].

**Fig. 2:**
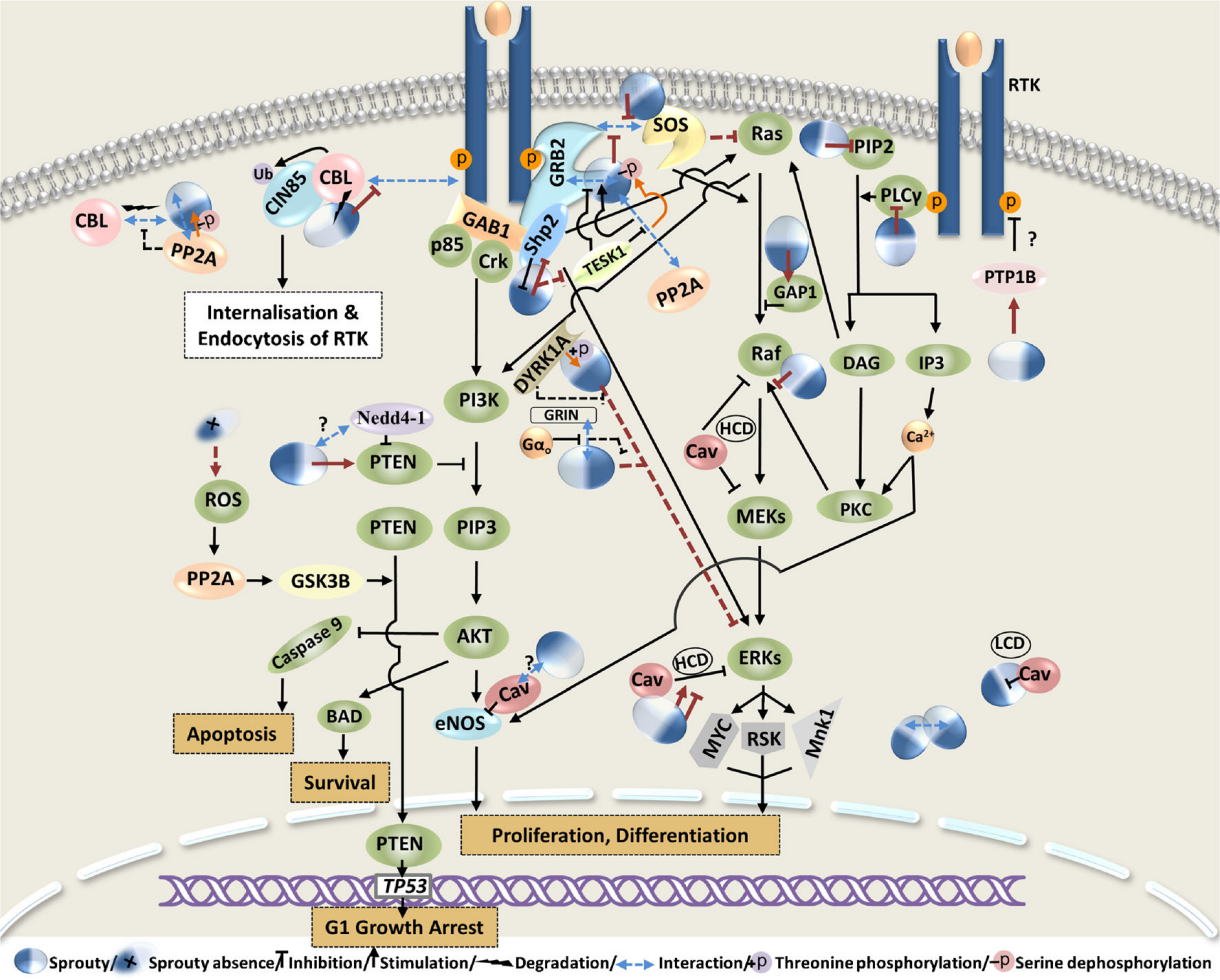
Schematic illustration of the Sprouty-mediated regulation of cell proliferation, differentiation, and survival irrespective of the Sprouty isoform and cell type. Sprouty activity is resulted from or regulated through interaction with a number of players. This interaction impacts functionality of ERK and other signaling pathways. Sprouty binds *c-Cbl* and *CIN85* and sequestrate c-Cbl to augment and prolong RTK signaling by inhibiting receptor endocytosis. This mechanism has been implicated in cell differentiation. E3 ubiquitin ligase c-Cbl, on the other hand, binds and induces degradation of Sprouty to restrict ERK activation. Sprouty has also been shown to interact with different phosphatases. It increases active contents of *PTEN* to mediate antiproliferative actions by inhibiting Akt activation. PTEN is also phosphorylated and accumulated in the nucleus in response to the Sprouty deficiency to induce p53-mediated growth arrest independently of its phosphatase activity. It is likely that the proto-oncogenic potential of NEDD4 is resulted in part from its ability to ubiquitinate both Sprouty and PTEN, resulting in unchecked activation of Akt. Sprouty also increases PTP1B content. However, there is no evidence of direct interaction between Sprouty and PTP1B in RTK dephosphorylation. Phosphatases *PP2A* and *SHP2* differentially regulate the Sprouty activity. Although PP2A potentiates Sprouty binding to Grb2 and thus positively regulates Sprouty by serine dephosphorylation, SHP2 promotes dissociation of Sprouty from Grb2 through tyrosine dephosphorylation and checks Sprouty inhibition of ERK. Moreover, interaction between Sprouty and kinases yields different outcomes. *DYRK1A* is considered a negative regulator of the Sprouty activity by threonine phosphorylation. *TESK1* interferes with Sprouty/Grb2 interaction as well as with Sprouty serine dephosphorylation by PP2A, thereby attenuating Sprouty functioning. Sprouty isoforms also exhibit differential cooperativity with *Cav-1* to repress growth factor activation of ERK. At low cell density, however, Cav-1 inhibits the Sprouty function. Sprouty is a general inhibitor of *PLC-*dependent signaling and inhibits various *PKC* upstream and downstream signals, including *PIP2* hydrolysis. Sprouty is an interacting partner of the *Gα*_*o*_ */GRIN* pathway. GRIN modulates Sprouty repression of ERK by binding and sequestering Sprouty. Activated Gα_o_, on the other hand, promotes inhibition of ERK via interacting with GRIN and releasing Sprouty. Finally, interaction among the Sprouty isoforms is a mechanism through which oligomers with more potent activity can form. *Cav* Caveolin-1; *c-Cbl* canonical Casitas B-lineage lymphoma; *CIN85* Cbl-interacting protein of 85 kDa; *DYRK1A* dual-specificity tyrosine-phosphorylated and -regulated kinase 1A; *Gα*_*o*_ G protein α_o_; *GRIN* G protein-regulated inducer of neurite outgrowth; *miR-21* microRNA 21; *Mnk1* MAPK-interacting kinase 1; *NEDD4* neural precursor cell expressed, developmentally down-regulated 4; *PAPC* paraxial protocadherin; *PIP2* phosphatidylinositol-4,5-bisphosphate; *PKC* Protein kinase C; *PLC* phospholipase C; *PP2A* protein phosphatase 2A; *PTEN* phosphatase and tensin homolog; *PTP1B* protein tyrosine phosphatase 1B; *RTK* receptor tyrosine kinase; *SHP2* Src homology-2 containing phosphotyrosine phosphatase; *Siah2* Seven in Absentia homolog 2; *TESK1* testicular protein kinase 1; *HCD* high cell density; *LCD* low cell density. In this figure, C- and N-terminus of the Sprouty molecule symbol are shown in *white* and *blue*, respectively. *Red lines* indicate the Sprouty effect, with *dashed lines* representing indirect influence. *Question marks* refer to postulated, but not proven, interactions

**Fig. 3:**
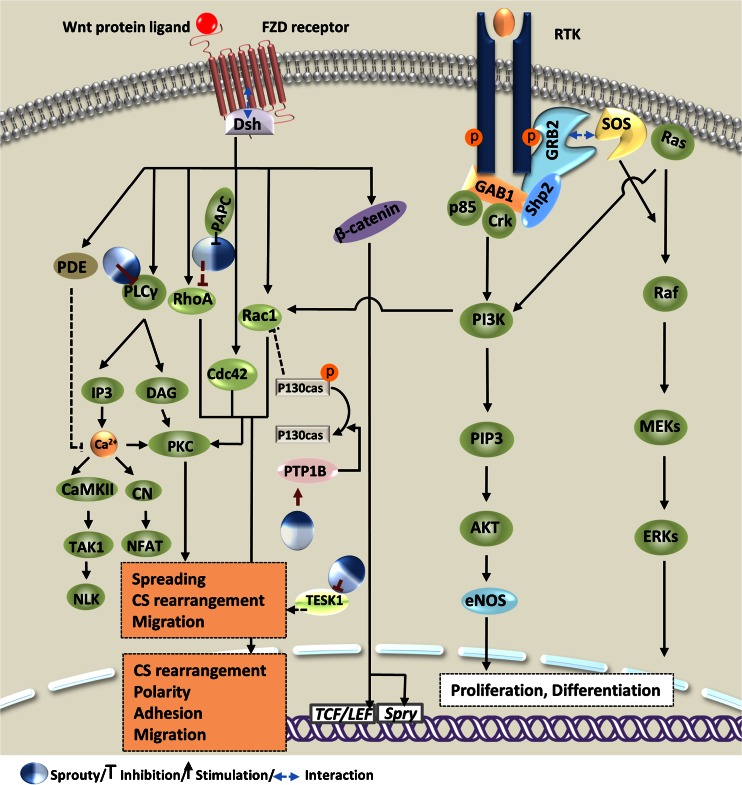
Schematic illustration of the Sprouty-mediated regulation of cell migration, adhesion, and cytoskeletal rearrangement irrespective of the Sprouty isoform and cell type. Sprouty is shown to interact with phosphatases. It increases active contents of *PTP1B* to mediate its antimigrative action by inhibiting activation of Rac1. Sprouty inhibits the kinase activity of *TESK1* that plays a critical role in integrin-mediated actin cytoskeletal reorganization and cell spreading. Sprouty is a general inhibitor of *PLC*-dependent signaling and inhibits various *PKC* upstream and downstream signals. Protocadherin *PAPC* implicated in modulating beta-catenin-independent Wnt-signaling has been suggested to mediate its regulatory effect by binding and sequestering Sprouty. *FZD receptor* Frizzled receptor; *PAPC* paraxial protocadherin; *PKC* Protein kinase C; *PLC* phospholipase C; *PTP1B* protein tyrosine phosphatase 1B; *RTK* receptor tyrosine kinase; *TESK1* testicular protein kinase 1; *CS rearrangement* cytoskeletal rearrangement. In this figure, C- and N-terminus of the Sprouty molecule symbol are shown in white and blue, respectively. *Red lines* indicate the Sprouty effect

In conclusion, the contributory role of the Sprouty downregulation in carcinogenesis and/or tumor progression and metastasis in the context of the breast [21], prostate [110, 135, 140, 147], liver [66, 71, 155], lung [31, 52, 56, 144, 157, 158], colon [13, 137], melanoma [95, 165, 166], B-cell lymphoid [67, 172], and thyroid [139, 173] cancer is documented. This contribution is an apparent reflection of the critical role of Sprouty in regulation of cellular processes central to the development, progression, and dissemination of malignant conditions, including cell proliferation, migration, invasion, transformation, and survival (Table 2). Mechanistically, Sprouty regulates cell behavior through modulation of the ERK activation along with interaction with a wide range of players and ultimate involvement of other regulatory mechanisms and cellular pathways as depicted in Figs. 2 and 3. Nevertheless, context-dependent contribution of Sprouty to cancer tumorigenicity and metastatic potential has also been reported in colon cancer [68, 69], as well as in RAS mutated fibrosarcoma [141] and rhabdomyosarcoma [70] as a result of E-cadherin repression and ensuing inhibition of the adhesive epithelial phenotype [69], upregulation of c-Met [68], and a concomitant RAS mutation [70, 141].

## Sprouty in cancer: Complexity and controversy

Under physiological conditions, as detailed earlier, Sprouty-mediated regulation is complex and multifaceted. Despite the initial understanding of Sprouty as a negative regulator of ERK, it is now evident that Sprouty has targets beyond ERK and functions, in concert with a variety of interacting molecules, in a cell- and context-dependent manner. Sprouty is differentially expressed by various normal cells not only during development, but also in adult organs in a tissue-specific or ubiquitous manner. Moreover, different Sprouty isoforms exhibit divergent regulatory functions. On this basis, it is not surprising that role of Sprouty in malignant conditions, where physiological homeostasis is altered in favor of neoplastic growth and progression, is fraught with intricacy and controversy. As discussed throughout this article, attempts have been made to shed light on unknown aspects of this story. In sum, our current knowledge indicates that the Sprouty’s implication in cancer, similar to its role under normal circumstances, is cell type- and context-dependent. Although deregulation of the Sprouty genes can indicate a general aspect of the Sprouty status in a given cancer, this needs to be interpreted in relation to the gene expression at the protein level and pertinent functional outcomes. A rewired genetic network with the involvement of Sprouty and ERK signaling apparently promotes tumorigenesis. However, the Sprouty gene association with tumor susceptibility or resistance may not be necessarily associated with a consistent phenotype *in vivo* due to somatic alterations. Thus, a combination of genetic and gene expression analysis has been recommended to complement genetic association methods for identification of susceptibility or resistance factors [165]. The expression of the Sprouty proteins, on the other hand, might be variably altered during tumorigenesis based on the pathogenic mechanism involved. Therefore, the expression pattern of Sprouty might be reflecting, for instance, a response to the mutant RAS-induced hyperactivation of ERK or, on the contrary, the epigenetic silencing of the Sprouty promoter. Moreover, Sprouty’s mode of action can be converted under malignant conditions. In the context of the RAS mutation, for example, Sprouty can function as an inhibitor [31, 52, 171] or facilitator [55, 68, 70, 141, ] of the tumor development and/or progression. This might be resulted in part from different functionality of the RAS isoforms [141].

Collectively, the expression pattern of Sprouty in different types of cancer is just a reflection of the primary or secondary deregulations incurred under specific circumstances. Since Sprouty is physiologically able to function as both a repressor and an activator of RTK signaling, its specific implication needs to be individually investigated in different cancers where its mode of action be evaluated in relation to the malignant cell behavior. In this regard, although investigation of the Sprouty gene aberrations and relevant oncogenic mutations can provide clues to the underlying mechanisms, evaluation of the effect of the Sprouty expression on cancer cell biology along with analysis of the clinicopathological relevance of the Sprouty deregulation will yield a better understanding of the Sprouty biology in a given cancer with potential application in the Sprouty-based approaches.

## Conclusion

Initially discovered as a growth factor antagonist with involvement in developmental processes, Sprouty is now recognized as a versatile modulator of ERK that also impacts other pathways to control crucial physiological processes in interaction with an increasing number of effectors, mediators, and regulators. Physiological functions of the Sprouty proteins are cell-specific and context-dependent. As such, Sprouty proteins are differentially induced in response to different growth factors and elicit divergent cellular responses. Moreover, transcriptional and post-translational regulation of the Sprouty content and activity provide spatiotemporal control of the Sprouty-mediated regulation. As regards the implication in malignancies, Sprouty has been the focus of research in a variety of studies for the past decade. Different patterns of the Sprouty deregulation have been reported in different cancers. As with normal cells, evidence shows that Sprouty in malignancies functions in a cancer cell-specific and context-dependent manner, hence its implication as a negative or positive regulator of the tumor progression. The presence of accompanying mutations of such oncogenes as RAS isoforms has also been shown to be an important determinant of the Sprouty’s deregulation and mode of action. To evaluate the role of Sprouty in a particular cancer with respect to putative clinical applications, in-depth investigation of the Sprouty’s expression and mode of action in relation to the malignant behavior of the cancer cell in the specific tumor microenvironment is warranted. This could give rise to a Sprouty-based stratification of individual patients where it serves as a biomarker of prognosis or treatment sensitivity as well as a focus of targeted strategies.

## Abbreviations

ARMS: Alveolar subtype of rhabdomyosarcoma
BCR: B-cell receptor
BDNF: Brain-derived neurotrophic factor
BPH: Benign prostatic hyperplasia
Cav-1: Caveolin-1
CBD: c-Cbl binding domain
c-Cbl: Canonical Casitas B-lineage lymphoma
ccRCC: Clear cell renal cell carcinomas
cDNA: Complementary DNA
CRD: Cysteine-rich domain
CREB: cAMP response element-binding protein
CtBP: C-terminal binding protein
dSpry: Drosophila Sprouty
DYRK1A: Dual-specificity tyrosine phosphorylation-regulated kinase 1A
EGF: Epidermal growth factor
EGFR: Epidermal growth factor receptor
ERK: Extracellular signal-regulated kinase
ERMS: Embryonic subtype of rhabdomyosarcoma
EST: Expressed Sequence Tag
FGF: Fibroblast growth factor
FRS2α: Fibroblast growth factor receptor substrate 2
Gα_o_: G protein α_o_
GDNF: Glial cell line-derived neurotrophic factor
GISTs: Gastrointestinal stromal tumors
GRIN: G protein-regulated inducer of neurite outgrowth
GSK3B: Glycogen synthase kinase 3 beta
HCC: Hepatocellular carcinoma
HCCP: Hepatocellular carcinoma with poorer outcome
HDM2: Human double minute 2
HER2: Human epidermal growth factor receptor 2
HGF: Hepatocyte growth factor
Hrs: Hepatocyte growth factor-regulated tyrosine kinase substrate
hSpry: Human Sprouty
KITLG: The ligand for the receptor tyrosine kinase c-KIT
LOH: Loss of heterozygosity
MAPK: Mitogen-activated protein kinase
MAP2K: MAPK kinases
MAP3K: MAPK-kinase kinase
MEF: Mouse embryonic fibroblasts
MEK: MAPK/ERK kinases
Mnk1: MAPK-interacting kinase 1
MPNST: Malignant peripheral nerve sheath tumors
mRNA: Messenger RNA
mSpry: Mouse Sprouty
MTC: Medullary thyroid carcinoma
myr-Akt: Myristoylated Akt
NEEC: Nonendometrioid carcinomas
NF1: Neurofibromin 1
NGF: Nerve growth factor
NSCLC: Non-small cell lung cancer
PDCD4: Programmed cell death 4
PDGF: Platelet-derived growth factor
PHD: Prolyl hydroxylase domain protein
PI3K: Phosphatidylinositol 3-kinase
PIN: Prostatic intraepithelial neoplasia
PIP2: Phosphatidylinositol 4,5-bisphosphate
PIP3: Phosphatidylinositol 3,4,5-trisphosphate
PKC: Protein kinase C
PKM2: Pyruvate kinase M2
PLC: Phospholipase C
PMA: Phorbol 12-myristate-13-acetate
PP2A: Protein phosphatase 2A
PPARγ: Peroxisome proliferator-activated receptor gamma
PTEN: Phosphatase and tensin homolog
PTP1B: Protein tyrosine phosphatase 1B
pVHL: von Hippel-Lindau protein
RMS: Rhabdomyosarcoma
ROS: Reactive oxygen species
RTK: Receptor tyrosine kinase
S1P: Sphingosine-1-phosphate
SHP2: Src homology 2-containing phosphotyrosine phosphatase
Siah2: Seven in Absentia homolog 2
SP1: Specificity protein 1
Spry1-4: Sprouty protein isoforms 1-4
SRM: Serine-rich motif
TCL1-tg: T-cell leukemia 1-transgenic
TCR: T cell receptor
TESK1: Testicular protein kinase 1
TGCT: Testicular germ cell tumors
TGFβ1: Transforming growth factor-beta1
TNF-α: Tumor necrosis factor-alpha
Tsg101: Tumor susceptibility gene 101 protein
uPA: Urokinase-type plasminogen activator
uPAR: Urokinase-type plasminogen activator receptor
VEGF: Vascular endothelial growth factor
WT: Wild type
WT1: Wilms tumor suppressor 1
xSpry: Xenopus Sprouty
zSpry: Zebra fish Sprouty
